# Supplementary material for: Are carbon emissions trading and green financial instruments synergistic? -Comprehensive quantitative research based on content analysis
Source: PLoS One. 2024 Mar 7;19(3):e0298601. doi: 10.1371/journal.pone.0298601 (PMC10919601; doi:10.1371/journal.pone.0298601)
Supplement: S1 Data — (ZIP) [file pone.0298601.s001.zip › Data/地级市Z_F工作报告.zip!/║╙─╧/σo╤⌠╩╨/└┤╫╘╣1⁄2╓┌║┼í╛┬φ┐╦╩2╛▌═°í┐_2015.docx]

各位代表：

现在，我代表市人民政府，向大会报告工作，请予审议，并请各位政协委员和其他列席人士提出意见。

一、2014年工作回顾

2014年是濮阳发展不平凡的一年，是迎难而上、持续赶超的一年。一年来，在省委、省政府和市委的正确领导下，全市上下认真贯彻党的十八大、十八届三中、四中全会精神和习近平总书记系列重要讲话精神，以开展党的群众路线教育实践活动和“一创双优”活动为动力，深入实施“4321”发展战略，坚持“1234”工作思路，积极应对复杂严峻形势，凝心聚力，克难攻坚，顶住了经济下行压力，较好地完成了市七届人大一次会议确定的目标任务。2014年的濮阳，经济稳中增效、转中跃升，社会和谐稳定、全面进步，城乡日新月异、焕然一新。

（一）经济发展持续赶超。经济规模不断壮大，速度效益同步提升。2014年生产总值完成1253.61亿元，增长10%，增速居全省第2位。规模以上工业增加值完成729.79亿元，增长12.8%，增速居全省第2位。一般公共预算收入70.4亿元，增长16.4%，增速居全省第7位。固定资产投资1115.8亿元，增长18.5%，增速居全省第13位。社会消费品零售总额412.29亿元，增长12.9%，增速居全省第4位。城镇居民人均可支配收入23767元，增长10.2%，增速居全省第1位。农民人均纯收入8828元，增长11.7%，增速居全省第2位。节能减排目标全面完成。12项经济指标增速进入全省第一方阵。

（二）产业实力明显增强。工业经济快速发展。深入实施工业强市战略“131”工程，育龙头、强集群、调结构、增效益。中原乙烯扩能改造等一批关乎濮阳产业发展的基础性、战略性重大项目取得新进展。丰利石化丙烯及联产芳烃、龙润化工甲醇制丙烯、双虎家居产业园等80个项目加快建设，惠成电子扩能改造、盛源科技顺酐、天能濮阳循环经济产业园一期等73个项目建成投产。中炜化工、双发实业等企业通过战略重组成长壮大。2014年全市新增规模以上工业企业93家，达到914家，实现主营业务收入3035.4亿元，利润262.3亿元，分别增长16.5%、18.6%。地方工业增长19.4%，传统产业增长8.5%，装备制造业增长16%，高新技术产业增长23.7%，工业结构进一步优化。产业集聚区快速发展，产业承载能力进一步增强，全市产业集聚区完成投资680.89亿元，增长36.3%；规模以上工业增加值增长19.6%。服务业快速发展。商务中心区和特色商业区加快推进。华龙区特色商业区名列全省十强。商贸物流业蓬勃发展，濮阳路港国际综合物流园、万利财富广场、亿洲乐活城市广场、正大商贸物流广场、天丰汽车城等一批商贸物流项目加快建设。华网传媒等一批电商企业迅速成长。金融服务业加快发展，台前村镇银行开业运营，市区农村信用社改制顺利推进。全市金融机构新增贷款82.31亿元，新增贷款存贷比93.9%。雪鸟实业、迈奇化学、蔚林化工等企业实现资本市场直接融资。全年资本市场融资90亿元，增长8.5%。文化旅游业发展迈出新步伐，旅游收入达到7.8亿元。河南省杂技“百戏奖”和全省杂技大赛落户濮阳，濮阳杂技品牌影响力进一步增强。成功承办了全国农村题材电视剧金牛奖颁奖典礼。2014年，服务业投资396.73亿元，增长20.8%。农业基础更加稳固。持续推进农业农村四项重点工作。实施高标准粮田“百千万”工程，新增有效灌溉面积3.2万亩、旱涝保收田面积2.9万亩，农业机械化率80%，粮食总产53.05亿斤，实现“十一连增”。农业产业化集群加快培育，全年新增省级以上龙头企业6家、现代农业示范园区9个、标准化养殖场56个，流转土地29.4万亩。植树2千余万株。积极培育新型农业经营主体，新增农民专业合作社678个，其中9家被评为国家农民合作示范社。实施黄河滩区扶贫开发五年攻坚行动，滩区群众脱贫致富步伐加快。范县张庄乡、陈庄镇8个村庄被省确定为滩区居民迁建试点。全年5万余农村贫困人口实现脱贫。

（三）发展优势不断积累。综合交通体系加快建设。晋豫鲁铁路建成通车，带来了煤资源，形成了出海通道。德商、南林等高速公路濮阳段加快建设。国道341、342、省道212等16条道路纳入省规划调整方案。新建改建农村公路1528.5公里。资源能源加快聚集。濮东500千伏输变电站建成投用，南水北调濮阳支线工程基本建成，文23战略储气库、龙丰热电2×60万千瓦机组、中原油田至开封输气管道等项目积极推进。2014年，我市被确定为国家级新型工业化示范基地、国家新能源示范城市、国家生物基材料产业集群建设示范市、省级生态文明先行示范区建设试点等，获得了政策支持，赢得了发展机遇。

（四）改革开放纵深推进。市、县政府机构改革全面启动，行政审批制度改革不断深化，压减审批项目140项，实施并联审批、网上审批，提高了行政效能。实施国企改革三年行动计划，7户企业完成改制任务。推动土地管理制度改革，农村集体建设用地和宅基地确权登记有序开展。实施财税体制改革，政府收支全部纳入预算管理，“营改增”范围进一步扩大。医药卫生、食品药品监管、户籍制度、公共服务等改革积极推进。民营经济活力不断增强，完成增加值786.01亿元，占GDP的比重达到62.7%。开放招商成效显著。实施招商引资“百日攻坚”行动，成功举办了中国石化技术装备大会暨首届中国（濮阳）石油装备展销会、中国羽绒行业发展论坛等活动，加强与央企、省企合作，全年签约亿元以上项目235个，实际利用省外资金175.3亿元，增长20%，位居全省第3位。河南商检濮阳办事处挂牌运行，设立海关分支机构取得新进展。深化油地战略合作，谋划了中原石化总厂扩能改造等重大产业项目，实施油田棚户区改造和市政工程，油地融合发展迈出新步伐。创新能力不断增强。新增高新技术企业7家，新建工程技术研究中心15家，重点实验室4家。一批企业通过产学研合作，建立产业技术联盟，实现了快速成长。高新技术创业服务中心被评为国家级科技企业孵化器。国家电光源检测中心、河南省羽绒质检中心建成投用，河南省家具质检中心、石油钻采设备及装备质检中心加快建设。我市被列为国家知识产权试点市、“全国质量强市示范城市”创建城市。国家农业科技园区建设取得新成效。

（五）城乡建设亮点纷呈。建设生态城市，倾力打造北方水城、中原绿都、生态宜居之城。示范区水库如期蓄水，龙山跃然成形，绿化全面启动，城区河道综合治理、“一河两路两园”和濮水公园改建形成景观，一个山叠翠屏、水映绿岸、路环绿带的大美濮阳呼之欲出。示范区卫都大街等18条道路建成通车，9座桥梁建成投用，建成区胜利西路、大庆路、黄河西路等城市道路改造完工，京开大道等10座公跨铁桥涵建成投用，城区道路更加畅通靓丽，群众由衷点赞。实施城市管网建设提升工程，扎实推进城中村、建成区旧片区、集贸市场改造。文化宫、图书馆等项目加快建设，教育园区初具规模，城市功能不断完善。强化城市管理，提升文明素质，顺利通过国家园林城市复检，成功创建为全国文明城市，濮阳的影响力和美誉度进一步提升。县域中心城市竞相发展。城市布局日趋合理，功能日益完善，品位不断提高，承载力、带动力显著提升。小城镇建设取得新进展。5个乡撤乡建镇。柳屯、濮城等9镇进入全国重点镇。全市城镇化率38.51%，较上年提高1.79个百分点。美丽乡村建设活动扎实推进，示范村、重点村建设取得明显成效。一批乡（镇）、村成功创建为省级生态乡（镇）、生态村。

（六）民生水平不断提升。十项重点民生工程圆满完成，社会事业全面发展。民生支出149.6亿元，占全市一般公共预算支出总量的80.1%，位居全省第2位。城镇新增就业7.28万人。企业退休人员基本养老金实现“十连增”。城镇居民医保和新农合人均财政补助标准提高到320元。医疗保险省内异地就医实现即时结算。80岁以上老人全部发放高龄津贴。开工建设各类保障性住房2.5万套，竣工1.05万套。完成农村危房改造6700户。新增集中供热面积150万平方米。回购更新公交车110辆。建设改造标准化中小学194所，改善农村薄弱学校办学条件20所。市新一高开工建设。与河南大学联办濮阳工学院取得突破，技师学院、杂技学校等加快建设。改建扩建乡（镇）卫生院20所，新建改建村卫生室885所。大力实施文化惠民工程，创作推出了杂技《白雪公主》、豫剧《清吏郑板桥》等一批精品剧目。全民健身活动蓬勃开展，竞技体育取得优异成绩。强化食品安全溯源监管，全市食品安全形势持续向好。开展“环保风暴”、“排雷行动”，实施“蓝天工程”、“碧水工程”，环境质量得到改善。做好信访稳定、安全生产、社会治安综合治理等工作，群众安全感和满意度继续保持全省前列，社会大局和谐稳定。

同时，人口计生、统计、民族宗教、发展研究、外事侨务、机关事务、残疾人工作、驻外联络、气象、防震减灾、人防、史志、档案等都取得了新成绩。国防教育、双拥工作扎实开展，驻濮解放军和武警部队在支持地方发展、维护稳定方面做出了积极贡献。

（七）政府自身建设不断加强。强化法制观念，坚持依法行政，法治政府建设迈出新步伐。完善责任、有序、高效的政府运行机制，行政效能不断提升。坚持民主科学决策，认真执行人大及其常委会决议和决定，支持政协参政议政，做好人大代表建议和政协委员提案办理工作。推进政务公开，自觉接受各方监督，广泛听取民主党派、工商联、无党派人士意见，支持工会、共青团、妇联等人民团体开展工作。加强廉政建设，落实“两个责任”，认真执行中央八项规定，“四风”突出问题得到有效整治。严格行政监察和审计监督，严肃查处违纪违法案件。强化政府投资监管，提升公共资源交易管理水平，着力营造为民、务实、清廉的政务环境。

各位代表！一年来，全市上下凝心聚力、矢志奋斗，以担当直面挑战，以实干推动发展，以执着成就事业。成绩来之不易，凝结着智慧和汗水，见证着责任和奉献。这些成绩是省委、省政府和市委正确领导的结果，是市人大、市政协大力支持的结果，是全市人民同心同德、拼搏进取的结果。在此，我代表市政府，向工作在各条战线的工人、农民、知识分子、干部、人民解放军指战员、武警官兵、公安民警以及关心、支持濮阳发展的各界人士致以崇高的敬意和衷心的感谢！

各位代表！我们清醒地认识到，我市经济社会发展和政府自身建设中，仍存在一些不容忽视的问题：一是经济下行压力依然较大，投资增速放缓，消费需求不足，受要素成本上升、融资难、融资贵等因素影响，部分企业生产经营困难。二是工业集群化水平不高，自主创新能力不强，战略性新兴产业亟待培育，第三产业发展滞后，农业持续增产、农民稳定增收难度加大，转型发展任务繁重。三是经济规模小，城镇化率低，基础设施相对薄弱，对外开放度不高，支撑发展的基础尚不稳固，聚要素、创优势的能力仍需提升。四是教育、医疗卫生、文化等民生领域欠账较多，生态环保等方面存在薄弱环节。五是政府职能转变不到位，一些干部的思想观念、工作作风、能力素质还不能完全适应新常态要求等。对此，我们一定高度重视，认真加以解决。

二、2015年工作总体要求和主要目标

2015年是全面深化改革的关键之年，是全面推进依法治国的开局之年，也是我市全面完成“十二五”规划和“二三五”赶超目标的收官之年。做好今年政府工作，责任重大，影响深远。综合判断，宏观经济形势依然复杂严峻，多重困难和挑战相互交织，今年的发展比去年难度更大，赶超任务更加艰巨。世界经济深度调整，复苏动力不足。我国经济进入新常态，速度变化、结构优化、动力转化，对赶超发展提出了更高要求。新常态蕴含新机遇，把握不好也会变成挑战。一方面，经济转型步伐加快、国家对基础设施和社会事业投入加大、消费拉动效果初显、创新驱动作用增强、产业格局持续调整、城镇化质量不断提升等，为我们在调整中“弯道超车”带来了新机遇，孕育了新希望。另一方面，我市处在工业化中期阶段，经济规模偏小、产业结构不优、科技创新能力不强，既需要完成工业化、城镇化的历史任务，又需要实现发展方式的根本转变；既需要壮大规模保持速度，又需要调优结构提高质量；既需要保持传统动力，又需要培育新型动力；既需要加快发展，又需要强化环境保护；既需要创新融资模式，又需要加强风险防范等。对此，我们必须高度重视，遵循规律，顺应形势，抢抓机遇，应对挑战，努力推动濮阳经济社会又好又快发展。

今年政府工作的总体要求是：深入贯彻党的十八大、十八届三中、四中全会精神和习近平总书记系列重要讲话精神，以“四个全面”战略布局为统领，认真落实河南省全面建成小康社会加快现代化建设战略纲要，按照市委经济工作会议部署，主动适应新常态，致力科学发展、赶超发展，保持态势、创新优势，着力加快产业转型升级，提高质量效益；着力提升基础支撑，增创发展新优势；着力改革开放创新，激发动力活力；着力建设生态城市，全面提升城市品质；着力实施民生工程，让人民共享发展成果；着力强化社会治理，促进社会和谐稳定。确保实现“二三五”赶超目标，为全面建成小康社会，加快建设富裕和谐美丽新濮阳奠定坚实基础。

今年经济社会发展的主要目标是：生产总值增长9%，在实际工作中努力争取更好结果；规模以上工业增加值增长12%以上；一般公共预算收入增长10%以上；固定资产投资增长20%；社会消费品零售总额增长13%；外贸出口总额增长8%以上；实际利用外资增长10%；城镇新增就业7万人；城镇居民人均可支配收入和农民人均纯收入分别增长10%、11%；居民消费价格指数涨幅控制在3%以内；人口自然增长率控制在6.5‰以内；节能减排实现省定目标。

实现上述预期目标，要把握好以下五个关键：一是必须把赶超发展作为首要任务。发展是第一要务，是解决一切问题的基础和关键。濮阳经济规模小、人均水平低，要与全国、全省同步实现全面小康，必须咬定发展不放松，高扬发展主旋律，致力科学发展、赶超发展。二是必须以新常态思维推动新发展。准确认识和把握经济新常态的阶段性和规律性，关乎发展快慢，关乎事业兴衰。我们必须以新常态的视角捕捉机遇、审视谋划发展，以法制理念、市场机制、创新动力推动发展，实现经济在中高速增长中迈向中高端水平。三是必须以深化改革激发动能。发展需要改革添动力，群众期盼改革出实效。我们必须把深化改革、扩大开放摆在更加突出的位置，统筹兼顾，抓重点、攻难点，以改革开放的新突破，为发展加力、让人民受益。四是必须以谋划运作赢得先机。注重顶层设计、战略谋划、科学运作，认真研究、准确把握国家政策走向，在重大事项确定、重大项目布局、重大产业发展等方面搞好精准对接，列专题、定专案、明确专人，抓好落实，保态势、创优势，赢得发展主动权。五是必须以保障改善民生作为发展根本。坚持人民至上，办好关系群众切身利益的身边事，办好事关群众冷暖安危的具体事，办好社会高度关注的焦点事，让人民群众得到更多实惠，生活得更富足、更舒心。

三、2015年重点工作

各位代表！今年的经济形势复杂严峻，发展任务异常艰巨，我们要始终把稳增长放在首位，直面困难不退缩、团结奋斗不懈怠，综合施策，扎实工作，谱写科学发展、赶超发展新篇章！

（一）着力做大做强工业。深入实施工业强市战略“131”工程，壮大规模，优化结构，上项目、延链条、保运行，推动工业质量和效益同步提升。今年，实施重点工业项目112个，年内完成投资359.9亿元。

壮大提升传统优势产业。化工产业，围绕石油化工、煤化工、盐化工“三化”融合链接，着力打造产值超千亿的集群，建设国家重要化工基地。壮大基础化工，谋划实施一批龙头项目，把油气、路煤等资源优势，转化为上游产业优势；发展精细化工，延链补链，向高端化、终端化方向发展。今年，丰利石化丙烯及联产芳烃等项目建成投产。中原大化气化岛、盛润集团甲醇制芳烃、绿能融创天然气处理等项目力争早日开工。中原乙烯扩能改造、中原石化总厂扩能改造等项目争取纳入国家、省“十三五”规划。加快实施君恒生物长链二元酸、沃森科技超高分子聚乙烯、班德路萜烯树脂等精细化工项目。石油装备制造产业，重点围绕石油钻探、采掘、管输延展产业链条，提升技术水平，着力打造中国石油装备制造基地。今年，重点推进中原特车、中原总机厂等企业与央企、省企开展战略合作，加快望联石油设备、一龙恒业石油钻采、博德石油设备、德瑞石油机械加工等项目建设。家具制造产业，围绕打造中国中部家具之都，加快产业集聚，着力抓好全友家私中部工业园、浙豫木业城、家具博览中心等项目建设。羽绒制品和食品加工产业，育龙头，树品牌，增强竞争力，重点推进双隆羽绒、永佳羽毛、众鑫羽绒、汇源肉羊养殖加工、康健休闲食品等项目建设。

大力发展战略性新兴产业。立足我市资源优势和产业基础，精心选择重点突破领域，着力培育化工新材料、生物基材料、新能源、高端装备制造、节能环保、生物医药等产业，抢占制高点，形成发展的战略支撑。今年，谋划推进乙烯向化工新材料方向发展。促进惠成电子、蔚林化工等新材料企业发展壮大。生物基材料和生物质能源产业，着力推进龙都生物聚乳酸纤维、盛润生物燃油、天人生物、聚乳酸感性树脂等一批重大项目建设。依托天能濮阳循环经济产业园，培育引进新型动力汽车等重大项目。加强与中石化新星公司合作，谋划推进地热资源综合开发利用产业化。注重集群引进，推进节能环保、生物医药等产业发展。

积极探索工业发展新举措。以新思维、新方式推动工业升级再造，构建现代工业体系。支持企业战略重组，鼓励企业以市场为纽带，利用资产、资源、品牌等优势，实施跨地区、跨行业联合、兼并，膨胀规模、提升层次，快速做大做强。鼓励引导制造业服务化，促进一批优势企业向研发、设计、物流、营销、品牌推广、系统集成方面发展。加快制造业和信息化的深度融合，推进“两化”融合试点示范工程建设，支持企业探索建设数字化车间和智能工厂，以信息化提升工业发展水平。注重品牌建设，着力培育一批名牌产品和品牌企业，打造区域品牌群，增强核心竞争力。

提升产业集聚区建设水平。加快推进综合服务、技术研发、检验检测、信息服务、物流等生产性服务平台建设，着力解决产业配套、金融支持、技术支撑、人力保障等突出问题。加快生活性服务配套建设，推进城市基础设施和公共服务向集聚区延伸覆盖，促进产城互动。完善集聚区基础设施，加快集聚区内村庄整合。优化产业空间布局，整合提升濮王产业园、濮州、户部寨化工专业园，规划建设濮阳市化工产业园。提升开发区化工产业层次，提高装备水平，延伸产业链条，着力打造科技含量高、经济效益好、绿色低碳循环的化工材料产业园区。

抓好工业经济运行。加强监测预警，强化要素保障，及时发现问题，综合施策，千方百计保运行、稳增长，防止工业经济出现大的波动。搞好企业服务，帮助上下游企业加强产销对接和协作配套，开拓市场。完善中小企业公共服务中心，为企业提供融资、人才培训、管理咨询、创业辅导、信息化推广等服务。

（二）大力发展第三产业。坚持生产性服务业和生活性服务业并重，现代服务业和传统服务业并举，着力培育产业高集聚、产出高效益、功能高复合、空间高密度、就业高容量的服务业集群，释放我市服务业发展巨大潜力。

构建现代市场体系。优化市场布局和结构，培育壮大五金建材、家居家装、农副产品、羽绒及制品等一批区域性专业市场。引导专业市场拓展物流、仓储、加工、金融、会展、信息、检验检测等增值服务。加快生产要素市场建设，建立健全人才、资源、能源等专业要素市场。加快二手车交易市场整合，规划建设循环经济产业园区，拉伸产业链条。

大力发展商贸物流业。推进开州城市综合体、恒丰中央广场、五洲商贸城、上亿广场等重大商贸项目建设。依托晋豫鲁铁路，规划布局大型物流园区，加快濮阳路港国际综合物流园、豫粮集团濮阳产业园等项目建设，打造区域性商贸物流中心。

发展金融服务业。发展地方金融机构，推进县（区）农村信用社改制组建农商行，清丰县、南乐县加快组建村镇银行。壮大金融主体，积极引进股份制银行。完善金融服务，加强银企对接，创新金融产品，扩大信贷投放。加快资金周转，优化信贷结构，降低融资成本，让更多的金融活水流向实体经济。力争2015年全市各级贷款新增85亿元以上。严厉打击非法集资活动，妥善化解防控各类风险。

发展文化旅游业。加快推进濮阳国际杂技文化产业园、东北庄原生态杂技文化旅游园区、濮阳杂技艺术学校建设，打造中国杂技艺术之都。谋划推进马庄遗址公园、瑕丘遗址公园、濮阳县古十字街保护利用、龙文化资源开发等项目，综合提升戚城遗址考古公园等。依托示范区水库龙山景观建设，开发环湖旅游线路，提升濮上园、绿色庄园品位，丰富内涵，形成区域旅游休闲新亮点。开发利用黄河、革命老区等资源，着力打造历史文化、黄河生态、红色经典等濮阳特色旅游品牌。

培育服务业新业态。大力发展电子商务，强化政策支持，规划建设电子商务产业园，培育一批电子商务示范企业，促进电子商务跨越发展。落实国家和省支持养老健康产业发展的政策，谋划实施一批项目，打造高端医疗和养老服务中心。积极培育云计算、大数据、物联网等业态。加快发展信息服务、文化创意、体育休闲、教育培训、服务外包等产业，培育新的经济增长点。

加快商务中心区和特色商业区建设。市商务中心区要进一步完善规划，加快路网及配套设施建设，着力引进商贸、金融、信息、企业总部等重大项目，促进服务业集群发展。各县“两区”要加快基础设施建设，完善配套功能，注重差异化、特色化发展，促进商业、旅游、文化及休闲功能融合，打造现代商业新亮点。华龙区特色商业区要在引进知名企业上下功夫，促进产业聚集，提升发展水平。

（三）加快推进新型城镇化。充分发挥城镇化的双拉动作用，以人的城镇化为核心，促进产业集聚、人口集中，形成市域中心城市、县城、小城镇联动发展的良好格局，着力构建现代城镇体系。

推进农业转移人口市民化。落实中央、省关于户籍制度改革、公共服务、社会保障等政策，研究制定附着在户籍上的教育、医疗、就业等配套改革措施，有序推进农村转移人口、棚户区和城中村居民落户城镇。对未落户的农业转移人口建立以居住证为载体的基本公共服务提供机制，切实保障进城农民工在农村的原有权益，消除农民进城的后顾之忧。

做大做强中心城市。以建设区域性中心城市为目标，综合提升建成区，加快建设示范区，完善功能，提升品位，提高城市的吸引力和辐射力。

加快生态城市建设。做活水、做足绿、做优文化，打造北方水城、中原绿都、生态宜居之城。打造生态水系，全面完成城区河道综合治理，结合实施引黄入冀补淀工程，建设生态景观廊道。打造生态绿系，全市完成植树2000万株以上。完成大广高速城区段绿化带、晋豫鲁铁路防护林带、黄河路东延绿化带等廊道绿化项目，推进城区道路绿化建设提升。开展示范区水库龙山绿化志愿行动，高标准完成示范区年度绿化任务。突出文化特色，注重将历史文化元素融入城市建设。

完善基础设施。做好濮阳市第四轮城市总体规划修编。完善城市路网。开工建设示范区龙泽大街、卫都大街东延、文明路北延、商鞅路、澶水路等道路。实施建成区金堤路北延、化工一路南延等工程。启动昆吾路跨铁路立交桥建设工程。完善城市地下管网。开展地下管廊建设试点，改造供热、供水、燃气管网50公里。推进城区东部、西部污水管网建设，实施雨污分离工程。完善公共配套设施。完成濮阳市汽车客运总站迁建。完成市第一污水处理厂改造，开工建设市第二污水处理厂二期工程，投入运行市第三污水处理厂。建成市第三水厂。新建改造一批城区公厕、垃圾中转站、雨污水泵站。加快示范区村庄迁建和东、西两湖社区建设。推进建成区戚城村、东干城、祁家庄等25个城中村改造，推进市电影公司、张庄闸管理处等4处片区改造。完善城区商贸市场。加快天龙市场、金宇市场、中原市场、劳动市场、大庆市场、油田商贸中心改造，新建一批农贸市场。强化城市精细化管理，建立长效机制，营造整洁、规范、有序的城市环境。

抓好县城和小城镇建设。县域中心城市要加快推进道路、供排水、供气供热、污水处理等基础设施建设，完善公共服务设施，增强教育、医疗等基础保障能力，提升品位，优化环境，着力打造实力县城、活力县城、魅力县城。推进濮阳县城、清丰县城、濮东组团与中心城区的对接融合，形成“一中心、三组团”协调互动发展格局。小城镇要强化基础，完善配套，绿化、亮化、美化、管理并举，着力建设特色小城镇。重点抓好33个建制镇综合提升，争取3个镇列入省100个重点镇建设示范工程。同时，贯彻落实好国家、省相关政策，促进房地产健康发展。

（四）积极推进农业现代化。坚持稳粮增收、提质增效、深化改革，加快转变农业发展方式，夯实“三农”基础。

提高粮食综合生产能力。实施高标准粮田“百千万”工程，完成30万亩高标准粮田建设任务。推广先进技术，提高机械化水平。加强农业综合开发，确保粮食总产稳定在50亿斤以上。

优化农业产业结构。大力发展都市生态农业和特色高效农业。加快国家现代农业园区建设，规划建设示范区现代农业园区，培育引进龙头企业，突出科技引领、创意创新，发展林果采摘、苗木花卉观赏、生态度假等特色都市农业。各县（区）规划建设一批为城市服务兼具休闲观光、生态体验等功能的现代农业园区。提高农业综合效益，新增设施农业7000亩，新建百亩以上现代农业园区6个，新建苗木花卉和经济林基地7万亩，新改建标准化养殖场50个以上。加快现代农业产业化集群培育，大力发展农副产品加工业，争取2个以上产业化集群进入省级产业化集群行列，新增产值超亿元龙头企业4家。推广农业标准化生产，确保农产品质量安全。推进沼气工程建设，高标准建成5座大型沼气工程和20座联户沼气工程，发展生态循环农业。

抓好滩区扶贫开发。实施好黄河滩区扶贫开发五年攻坚行动。重点推进道路、水利、电网等生产性基础设施建设。大力发展特色产业，推广高效种植和绿色养殖，发展农副产品深加工和劳动密集型产业。抓好订单、定向、定岗技能培训，推动劳动力转移就业，拓宽滩区群众致富渠道。扎实推进范县张庄乡、陈庄镇居民迁建试点工程。积极申报第二批省级迁建试点。

建设美丽乡村。坚持“五规”合一，科学编制新农村建设规划。按照“通、净、绿、亮、文”的标准，巩固提升7个示范乡（镇）、210个示范村和环境整治重点村综合整治成果，再启动7个示范乡（镇）、80个示范村、150个环境整治重点村综合治理。建立完善农村生活垃圾村收集、乡（镇）运输、县处理机制。加强历史文化传统村落和民居保护。

推进农村改革。大力发展新型经营主体，加快培育新型职业农民，支持家庭农场、农业专业合作组织、种养大户发展壮大。强化农业公共服务和社会化服务，支持开展农业保险。做好农村土地承包经营权和农村宅基地使用权确权。深化集体林权制度改革。探索建立农村产权交易市场，促进农村土地经营权有序流转，实现适度规模经营。

（五）加强基础能力建设。谋划推进一批事关濮阳长远发展的大事要事，聚集资源要素，强化战略支撑，增创区域竞争新优势。

加快现代交通体系建设。着力将濮阳区位特点转化为区位优势。铁路方面，完善晋豫鲁铁路通道配套功能，加快郑濮济快速铁路前期工作，争取濮阳至潢川铁路、邯郸经濮阳至菏泽铁路列入省“十三五”规划。高速公路方面，开工建设范台梁高速豫鲁省界至范县段，完成德商高速范县段、南林高速豫鲁省界至南乐段，推进濮阳至湖北阳新高速前期工作。干线公路升级改造方面，启动省道101濮阳县王助至范县杨集段升级改造，抓好国道341濮阳段、省道307濮城东至八公桥段升级改造，加快省道215改造项目前期工作。推进台前孙口黄河公路大桥等项目。新建改建农村公路500公里，改造危桥3880延米。

强化能源资源保障能力。推进国家综合能源物流中心和交易中心建设。开工建设文23战略储气库。建成投用中原油田至开封输气管道。推进日照经濮阳至洛阳3000万吨输油管道、丰利石化至东明原油管道、濮城至浚县成品油管道建设。推进濮阳经范县至台前、南乐至寺庄天然气管道建设。推进市煤炭储运中心、濮阳石化能源物流仓储基地建设。开工建设龙丰热电2×60万千瓦机组、盛润集团背压发电机组、市生活垃圾焚烧发电、生物质能热电、光伏发电等项目。完成台前220千伏输变电工程。谋划推进台前2×100万千瓦机组项目。完成农村电网改造年度任务。

推进重大水利工程建设。年内开工建设引黄入冀补淀工程，濮上河竣工通水，全面完成南水北调濮阳支线工程。完成全国小型农田水利重点县项目年度工程，实施渠村、南小堤两个大型灌区和于庄中型灌区配套改造工程，实施滩区灌排工程，增强水资源保障能力。

加强信息基础设施建设。推动城乡光纤覆盖和普及提速，优化4G网络，启动城区无线局域网全覆盖工程，完善数字城管系统，着力建设智慧城市。

争取政策支持。切实做好“十三五”规划编制，谋划全局性重大项目、重大工程、重大生产力布局，争取列入国家、省发展规划，赢得发展先机。积极创建国家生态文明先行示范区，用足用好资源型城市转型试点市、国家级新型工业化示范基地等金字招牌，形成政策优势，促进赶超发展。同时，强化项目支撑。今年，着力实施好318个省、市重点项目，年内完成投资860亿元以上。对列入省扩大有效投资“双十”计划的374个项目，按照计划节点组织实施，确保完成年度建设任务。对接国家和省产业政策，再谋划实施一批重点项目，切实增强发展后劲。

（六）全面深化改革开放。坚持把改革开放作为激发动力活力的根本举措，推动经济持续快速健康发展。

深化改革。全面完成市县政府机构改革。加快行政审批制度改革，继续清理压减行政审批事项，取消非行政许可审批。规范行政审批行为，开展行政审批标准化建设，加强事中事后监管。加大国有企业改革力度，分类施策，通过整合重组、依法破产等形式，焕发优势国有企业生机活力，实现劣势国有企业有序退出。完善国资监管体制，提高资本运作水平，实现保值增值。注重引进央企、省企及民企参与改革，积极发展混合所有制经济，增强企业活力。深化财税管理体制改革，实行政府全口径预算管理，扩大“营改增”试点，推进政府购买服务。加快医药卫生体制改革，推进市、县公立医院综合改革，引导支持社会办医。按照统一部署，扎实推进事业单位分类、公安体制、供销管理体制、不动产登记、公积金等改革。大力发展民营经济，完善扶持政策，实施民营企业“百千万成长工程”，推动民营经济扩量提质。

扩大开放。主动融入国家“一带一路”战略，对接郑州航空港经济综合实验区，依托晋豫鲁铁路，打造出海大通道；加快国检濮阳办事处检验检疫综合技术用房建设，争取设立海关分支机构，打造濮阳“无水港”，形成大开放新格局。转变招商引资方式，以良好的环境、优质的服务、完善的配套构筑产业生态圈，吸引国内外客商。围绕培育战略性新兴产业、提升传统优势产业，绘制产业图谱和招商图谱，推行精准招商、以商招商、中介招商、集群招商，切实增强招商引资的针对性和时效性。今年，重点开展好光电子、羽绒及服饰加工、石油装备制造等产业15项专题招商活动。完善招商推进机制，成立产业招商小组，明确招商目标，大员上阵，高层调度，业长负责，部门协作，市、县（区）联动，强化考核激励，努力实现招商引资新突破。着力做好外经外贸工作，提升国家专用化工品、羽绒及服装制品等6个出口基地建设水平，力争创建省级文化产业出口基地，促进外贸转型升级。大力发展对外工程承包和劳务合作，鼓励支持中原油田、濮耐公司等企业开展跨国经营。加强与中石油、中海油、中机集团、华电集团、河南投资集团、省国控公司、河南能化等央企、省企开展战略合作，实施一批重大产业项目。

提升油地融合发展水平。油地双方携手共进，战略合作成果丰硕，进入了新的更高境界。我们要倍加珍惜油地双方肝胆相照、共赢发展的良好局面，立足当前，着眼长远，建立健全油地合作长效机制，谋划实施一批重大基础设施和产业项目，凝聚发展合力，共建美好家园。

（七）着力破解发展瓶颈。运用新常态思维，攻难点、破难题，促进科学发展、赶超发展。

突出创新驱动。着力增强科技创新能力，提升国家级科技企业孵化器建设水平，扶持一批国家和省、市公共技术研发设计中心、工程技术研究中心、重点实验室，促进企业与大型央企、科研院所、高等院校共建创新平台。加快创新主体培育，培育高新技术企业3家、省级节能减排科技创新示范企业2家、市级节能减排科技创新示范企业6家。实施专利技术产业化示范工程，培育专利技术示范企业5家。抢抓国家实施“互联网+”行动计划重大机遇，着力培育一批新兴产业和新兴业态。鼓励引导企业开展商业模式创新，带动生产组织方式、产品、工艺的创新，形成竞争新优势。完善科技创新体制机制，探索通过金融支持、创投引导、风险补偿、补助等方式促进科技创新，完善技术转移和成果转化服务机制。建立健全创新奖励机制，优化创新生态，厚植创新文化，鼓励大众创业、万众创新。对在重大科技专项、重点科技工程中作出突出贡献的领军人物和创新团队予以重奖，激发创新热情。加强人才培养与引进，建设一支高素质的创新人才队伍。

创新融资方式。推行政府与社会资本合作的PPP模式，建立项目库，向社会推介一批示范项目，吸引民间资本参与建设运营。主动对接省新型城镇化发展母基金，研究设立市、县子基金，破解城市基础设施和公共服务建设的资金制约。推进财政性涉企资金基金化改革，探索设立创业投资和产业投资基金，发挥基金引导作用，促进大众创业、新兴产业发展。推进企业上市融资。支持优势企业发行债券。发展壮大担保公司，探索设立“过桥还贷”资金，为企业发展提供支持。积极推进市建投、市经开、市国控等投融资平台公司化运作，壮大实力，提高投融资能力。加强与国开行、省水投等战略合作，为濮阳发展提供资金保障。今年，完成资本市场融资95亿元。

节约集约利用土地。认真贯彻国家土地政策，加强耕地保护，做好永久基本农田划定和土地利用总体规划调整完善工作，科学划定永久基本农田红线、城市开发边界和生态保护红线。搞好土地综合整治，创新土地供应方式，扩大国有建设用地有偿使用范围，盘活存量、用好增量，有限指标保重点。做好城乡建设用地增减挂钩试点工作，加强对项目占补平衡指标的统筹协调，保障重大项目用地需求。

加强生态环境建设。深入开展污染防治，持续开展“环保排雷”专项行动，对不达标企业限期整改，整改不达标的予以关停。严格项目准入制度，确保新上项目环保达标。实施蓝天工程、碧水工程和城市河流清洁行动计划。严控颗粒排放，推进电力机组脱硫脱硝、城市燃煤锅炉改造，抓好黄标车治理、建筑工地及道路交通扬尘治理。加强空气、水质检测，实施17个空气、水质自动监测站建设改造。加强危险废弃物处理中心、医疗废弃物处理中心建设。大力发展循环经济，争创国家级可持续发展实验区，实施传统产业循环化改造，启动一批节能降耗和循环化改造项目，实现资源高效循环利用，努力构建绿色产业体系。

（八）切实保障改善民生。加大财政投入，实施好十项重点民生工程，大力发展社会事业，织密织牢民生保障网，增进人民福祉。

保持就业稳定增长。提升人才市场服务水平，做好高校毕业生、城镇困难人员、退役军人等就业工作。完善公共服务体系，推进职业教育攻坚二期工程、全民技能振兴工程，提高劳动者就业创业能力。

促进城乡居民增收。城乡居民养老保险基础养老金标准每人每月提高20元，企业退休人员基本养老金水平提高10%。继续提高城乡低保、农村五保和优抚对象补助标准。完善最低工资标准调整机制。落实机关事业单位人员工资调整方案，实行乡（镇）工作补贴。强化欠薪治理，保障农民工合法权益。多渠道增加农民收入。

提升社会保障能力。推进社会保障一卡通。实施机关事业单位养老保险改革。实行城乡统一的独生子女父母奖励扶助制度和计划生育家庭特别扶助制度。实施临时救助，让身处困境者得到关爱。加强社会养老服务体系和县级社会福利中心建设，实施73所乡（镇）敬老院综合提升工程。健全农村留守儿童、妇女和老人关爱服务体系。建设市残疾人综合服务中心。建设各类保障性住房1.2万套，完成农村危房改造4050户。年内完成公交运营体制改革，回购127辆公交车。

办好人民满意教育。统一城乡义务教育学校公用经费标准。启动第二期学前教育三年行动计划和特殊教育提升计划。启动实施扩充城镇基础教育资源五年规划。调整城乡学校布局，改善城乡教育基础设施，创建190所义务教育标准化学校。实施“全面改薄”工程，对47所农村中小学校舍进行加固维修或拆除重建，改善义务教育薄弱学校基本办学条件。实施农村教师周转房专项工程。推进市一高新校区等项目建设。发展现代职业教育，从今年秋季起对全日制中职在校生全免学费。加快市杂技艺术学校、市高级技师学院建设。做好河南大学濮阳工学院筹建工作，争取年内实现招生。开工建设市委党校新校区。

让人民群众享受文化建设成果。巩固提升全国文明城市创建成果，认真践行社会主义核心价值观，做好人、行好事、立好心，提升城市文明程度和全民文明素质。强化公共文化服务，建成市工人文化宫、市图书馆新馆，开工建设市烈士陵园。完善县（区）公共文化服务设施，做好基层综合文化服务中心试点建设。实施广播电视户户通、舞台艺术送农民等文化惠民工程。加强网络文化建设和管理，净化网络空间。持续“扫黄打非”，抵制低俗现象。

提升群众健康水平。加快公共卫生和基本医疗服务体系建设，继续实施乡村医疗机构标准化建设攻坚行动，改扩建20所乡（镇）卫生院，新建改建600所村卫生室。抓好市人民医院病房楼、市红十字医院康复病房楼等医疗设施建设。新农合和城镇居民医保财政补助标准每人每年再提高60元，基本公共卫生服务经费人均财政补助标准提高到40元。完善新农合大病保险，全面推行城镇居民大病保险，实施疾病应急救助制度。开展爱国卫生运动，巩固提升国家卫生城市创建成果，确保通过国家复审。大力发展体育产业，广泛开展全民健身活动。

创新社会治理。完善基层民主科学决策、矛盾调解化解、服务群众和党风政风监督检查四项基础制度，夯实基层工作基础。推行重大决策社会稳定风险评估机制，推动社会矛盾调处由事后处置向事前预防转变。坚持访诉分离和法定途径优先原则，切实解决信访突出问题，推动全市信访形势和信访秩序持续好转。强化食品药品安全监管，推进食品安全溯源监管综合平台二期工程建设，落实生产经营者主体责任，严惩各类食品安全违法行为，提高群众满意度。强化安全生产监管，坚决遏制重特大事故发生。创新立体化社会治安防控体系，严厉打击违法犯罪活动。健全反恐工作机制，完善应急管理体系。实施“满天星”三期工程，加强平安濮阳建设，维护社会和谐稳定。

同时，继续加强国防教育和国防后备力量建设，支持驻濮解放军、武警消防部队、预备役部队、民兵和人民防空建设，深入开展双拥共建，巩固军政军民团结。支持工会、共青团、妇联等人民团体发挥桥梁纽带作用。做好民族宗教工作，发挥宗教界人士和信教群众在促进经济社会发展中的积极作用。统筹做好统计、工商、发展研究、外事侨务、机关事务、驻外联络、史志、档案、防震减灾、气象、邮政、通信等工作。

四、加强政府自身建设

建设法治政府。深入贯彻依法治国基本方略，强化法治意识，用法治思维和方式履行职责，按照法定权限和程序行使权力。坚持权由法授、权责法定，推行负面清单、权力清单、责任清单“三位一体”管理，法无授权不可为，法定职责必须为。健全政府决策机制，发挥智库作用，推行法律顾问制度，依法决策、科学决策、民主决策。严明政治纪律，严守政治规矩。自觉接受人大法律监督、政协民主监督、人民群众和新闻媒体的监督，认真落实人大决议，办理好人大议案、代表建议和政协提案。积极开展协商民主工作，广泛听取民主党派、工商联、无党派人士和各人民团体意见。加强政务信息公开，回应社会关切。落实行政执法责任制，严格执法监督，促进规范公正文明执法。畅通行政复议渠道，依法化解行政争议。完成“六五”普法任务。严格依法行政考核，把政府工作纳入法治轨道。

建设服务政府。转变政府职能，忠诚履职，知责有为，敬业奉献，勇于担当，把干事创业谋发展、促赶超、惠民生作为最大责任，多做打基础、利长远的事情，不做表面文章，不搞形象工程。改进服务方式，规范工作程序，推行一口受理、首问负责、限时办结、网上办理、并联审批等制度，提升行政效能。完善领导分包、观摩考评、现场办公、督导督查、项目调度等工作机制，强化台账管理，确保工作落实到位。强化行政问责，坚决纠正不作为、乱作为，坚决克服懒政、怠政，提高执行力和公信力，不断优化政务环境。

建设廉洁政府。加强惩防体系建设，落实“两个责任”，遵守廉洁自律各项规定，加大案件查办力度，保持反腐高压态势，坚决纠正行业不正之风，维护群众切身利益。深入贯彻中央“八项规定”精神，扎实开展“三严三实”专题教育，驰而不息纠正“四风”。坚持勤俭节约，从严控制“三公”经费，完成党政机关公务用车制度改革。健全行政权力运行制约和监督机制，加强和完善政府内部层级监督，强化监察、审计监督，严格政府投资监管，把权力关在制度的笼子里，套上责任的紧箍咒。加强反腐倡廉教育和廉政文化建设，弘扬清风正气，当好人民公仆。

各位代表！致力科学发展、赶超发展是我们的责任，社会和谐、人民幸福是我们的追求。让我们紧密团结在以习近平同志为总书记的党中央周围，在省委、省政府和市委的坚强领导下，凝心聚力，砥砺奋进，加快建设富裕和谐美丽新濮阳，为促进中原崛起河南振兴富民强省做出新的更大贡献！
